# Characterization of visual processing in temporomandibular disorders using functional magnetic resonance imaging

**DOI:** 10.1002/brb3.2916

**Published:** 2023-02-15

**Authors:** Daniel E. Harper, Kaundinya Gopinath, Jeremy L. Smith, Mia Gregory, Eric Ichesco, Sharon Aronovich, Richard E. Harris, Steven E. Harte, Daniel J. Clauw, Candace C. Fleischer

**Affiliations:** ^1^ Department of Anesthesiology Emory University School of Medicine Atlanta Georgia USA; ^2^ Department of Radiology and Imaging Sciences Emory University School of Medicine Atlanta Georgia USA; ^3^ School of Biological Sciences Georgia Institute of Technology Atlanta Georgia USA; ^4^ Chronic Pain and Fatigue Research Center, Department of Anesthesiology University of Michigan Medical School Ann Arbor Michigan USA; ^5^ Department of Oral and Maxillofacial Surgery and Hospital Dentistry University of Michigan Michigan Medicine Ann Arbor Michigan USA; ^6^ Department of Biomedical Engineering Georgia Institute of Technology and Emory University School of Medicine Atlanta Georgia USA

**Keywords:** chronic pain, fMRI, MRI, TMD, visual processing

## Abstract

**Background and Purpose:**

Many patients with chronic pain report hypersensitivity not only to noxious stimuli, but also to other modalities including innocuous touch, sound, and light, possibly due to differences in the processing of these stimuli. The goal of this study was to characterize functional connectivity (FC) differences between subjects with temporomandibular disorders (TMD) and pain‐free controls during a visual functional magnetic resonance imaging (fMRI) task that included an unpleasant, strobing visual stimulus. We hypothesized the TMD cohort would exhibit maladaptations in brain networks consistent with multisensory hypersensitivities observed in TMD patients.

**Methods:**

This pilot study included 16 subjects, 10 with TMD and 6 pain‐free controls. Clinical pain was characterized using self‐reported questionnaires. Visual task‐based fMRI data were collected on a 3T MR scanner and used to determine differences in FC via group independent component analysis.

**Results:**

Compared to controls, subjects with TMD exhibited abnormally *increased* FC between the default mode network and lateral prefrontal areas involved in attention and executive function, and *impaired* FC between the frontoparietal network and higher order visual processing areas.

**Conclusions:**

The results indicate maladaptation of brain functional networks, likely due to deficits in multisensory integration, default mode network function, and visual attention and engendered by chronic pain mechanisms.

## Graphical abstract

Using a visual task‐based paradigm combined with functional magnetic resonance imaging, we observed individuals with temporomandibular disorders (TMD) and chronic pain exhibited abnormally increased functional connectivity between the default mode network and lateral prefrontal areas involved in attention and executive function compared to pain‐free healthy controls (HC). Impaired functional connectivity in subjects with TMD was also observed between the frontoparietal network and higher order visual processing areas.

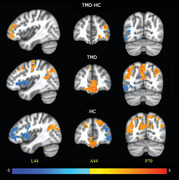

## 1 INTRODUCTION

Chronic pain is a major public health challenge, exacerbated to some extent by the lack of effective treatments. Research has identified peripheral and centralized, or *nociplastic*, contributions to chronic pain, including temporomandibular disorders (TMD) (Harper et al., 2016; Scholz, 2014). TMD has a lifetime prevalence of ∼10–12% (Manfredini et al., 2011) and is one of many chronic overlapping pain conditions including fibromyalgia and irritable bowel syndrome; however, much less is known about central pain processing in TMD compared to other conditions. Nociplastic pain mechanisms can include changes in brain structure, function, and metabolite concentrations (Eller‐Smith et al., 2018; Harfeldt et al., 2018; Harper et al., 2016). Additionally, demonstration of hypersensitivity to nonsomatosensory stimuli in chronic pain suggests the presence of generalized, central mechanisms of sensory amplification (Geisser et al., 2008; Harte et al., 2016; Hollins et al., 2009; Kmiecik et al., 2022).

The mechanisms underlying discomfort evoked by nonsomatosensory stimuli in TMD are unclear, despite reports of multisensory hypersensitivity (Greenspan et al., 2013; López‐Solà et al., 2017; Martenson et al., 2016; Phillips & Clauw, 2011; Schrepf et al., 2018; Ten Brink & Bultitude, 2022; Ten Brink et al., 2021). Differences in visual‐evoked functional connectivity (FC) and brain activation have been observed in other pain conditions (Harte et al., 2016; Cottam et al., 2018) and may explain the multisensory sensitivity in TMD (Shen et al., 2019). Visual stimulation is not an irritant for all TMD patients, but it is likely more unpleasant in a subset of individuals with nociplastic (as opposed to peripheral nociceptive) pain. Therefore, the goal of this pilot study was to identify differences in the brain networks of TMD patients, compared to pain‐free controls, during an unpleasant visual functional magnetic resonance imaging (fMRI) paradigm (i.e., a visual checkerboard stimulus) to uncover possible nociplastic pain‐associated mechanisms that would not be evoked by noxious stimulation of a painful site.

## 2 METHODS

### 2.1 Study design and subjects

This study was approved by the local Institutional Review Board. All subjects provided written informed consent prior to participation. Patients with painful and clinically diagnosed TMD were recruited from Oral and Maxillofacial Surgery/Hospital Dentistry. Pain‐free controls were recruited from the local community and were healthy without history of chronic pain. Exclusion criteria for both cohorts were severe physical impairments (e.g., bilateral amputation); medical conditions (e.g., autoimmune diseases, cancer); severe psychiatric illnesses; opioid, tobacco, or hormone use; or pregnancy. One subject was excluded due to challenges with data acquisition and poor image quality. The final cohort consisted of 10 subjects with TMD (*n* = 9 female), 18–49 years old (mean ± standard deviation [SD] = 32 ± 10 years), and 6 female pain‐free healthy controls (HC), 19–51 years old (31 ± 12 years). Subjects completed pain questionnaires followed by MRI acquired either the same or following day.

### 2.2 Clinical pain metrics and visual unpleasantness

The American College of Rheumatology's 2011 Preliminary Diagnostic Criteria for Fibromyalgia, which includes Symptom Severity and Widespread Pain Index (WPI) subscales, was administered (Wolfe et al., 2011), with higher scores (range: 0–31) (Wolfe et al., 2011) indicating likely nociplastic pain. The current TMD symptoms questionnaire from the TMD Research Diagnostic Criteria (RDC; 2002) was used to assess current face pain (Dworkin & LeResche, 1992). The short form of the Brief Pain Inventory (BPI) was used to assess pain severity and interference. The Pennebaker Inventory of Limbic Languidness (PILL) was used to measure somatic awareness or hypervigilance (Pennebaker, 1982). Immediately after the visual scan, participants were asked to rate the perceived unpleasantness of the visual stimulus on a scale from 0 to 100, where 0 means “not at all unpleasant” and 100 means “the most unpleasant sensation imaginable.” Mann–Whitney *U* tests were used to compare age, clinical pain metrics, and perceived visual unpleasantness between groups.

### 2.3 MR data acquisition

**FIGURE 1:**
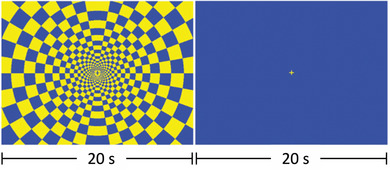
Visual paradigm used during fMRI. The visual task paradigm included six “on–off” cycles. The “on” block (left image) displayed an 8 Hz flashing checkered visual task for 20 s followed by an “off” block (right image) displaying a blue screen with a yellow crosshair in the center for 20 s.

MR data were acquired on a 3T whole‐body MR scanner (Signa Discovery MR750, GE Healthcare, Chicago, IL) with a 32‐channel head coil. T1‐weighted images were acquired using a spoiled gradient recalled echo sequence (repetition time [TR] = 650 ms, echo time [TE] = 3.7 ms, flip angle = 8°, voxel size = 1.0 × 1.0 × 0.8 mm^3^, field of view [FOV] = 256 × 256 mm^2^, 166 slices). fMRI data were acquired using a multiband gradient echo pulse sequence (TR = 1200 ms, TE = 30 ms, flip angle = 70°, FOV = 210 × 210 mm^2^, 2.4 mm × 2.4 mm in‐plane resolution, 51–2.5 mm thick slices, multiband acceleration factor = 3). The visual paradigm included six cycles of 20 s “on” (8 Hz flashing checkerboard) and “off” (static crosshair) blocks (Figure 1). This same paradigm was used in Harte et al. (2016) to elicit unpleasantness and measure brain activity in fibromyalgia patients.

### 2.4 MR data preprocessing

Functional data were preprocessed using SPM12 and employing well‐established pipelines (Anteraper et al., 2021; Glasser et al., 2013; Gopinath et al., 2015). The fMRI voxel time‐series data were temporally shifted to account for differences in slice acquisition times, and three‐dimensional volumes were registered to a base volume. These data were then corrected for physiological noise (respiratory and cardiac) with the well‐established RETROICOR (Glover et al., 2000) technique, and spatially normalized to the MNI‐152 template. Outlier identification was performed by flagging acquisitions with fMRI time‐series signal variation >5 SDs or frame‐to‐frame displacement >0.9 mm. First quartile, third quartile, and maximum motion across all subjects and scans were 0.17, 0.28, and 0.57 mm, respectively.

### 2.5 Group independent component analysis

The preprocessed fMRI data from all subjects were spatially smoothed with an isotropic Gaussian filter (full width at half maximum = 6 mm) for independent component analysis (ICA) analysis. Group ICA (GICA) was performed on temporally concatenated data from all subjects (TMD and HC) using the well‐established GIFT software (Calhoun et al., 2001). This ICA algorithm decomposes the concatenated fMRI time‐series data into group‐level independent components (ICs), which are composed of maps (component strength expressed as t‐scores), and their corresponding IC time‐courses. The ICA decomposition maximizes the independence between the spatial maps of different ICs, while not constraining the form of the time‐courses. Subject‐level ICs corresponding to the group ICs were obtained through the ICA back‐projection technique (Calhoun et al., 2001). The group‐level GICA spatial maps were visually examined for artifactual components representing draining veins, physiological noise, ventricular signal, or motion, using a well‐established approach (Allen et al., 2011; Cetin et al., 2014). The nonartifactual ICs constitute different brain functional networks. Voxel‐wise maps of group differences in FC to ICA‐derived brain functional networks were obtained with independent‐samples *t*‐tests on corresponding spatial maps. These group *t*‐test results were clustered, and the family‐wise error rate‐controlled significance (*α*) was computed for the given cluster detection threshold *t*‐score (*p* < .05) using Monte Carlo (MC) simulations of the process of image generation. Simulations were controlled for the estimated spatial autocorrelation function of regression residuals, intensity thresholding, masking, and cluster identification using the 3dClustSim program in AFNI (Cox, 2019; Gopinath et al., 2018).

## 3 RESULTS

Comparisons of clinical pain metrics and age are presented in Table 1. Current face pain, symptom severity, WPI, fibromyalgianess, PILL, and BPI were all significantly higher in the TMD cohort compared to HC (*p* ≤ .05). Despite perceived visual unpleasantness being somewhat higher in TMD patients (mean = 43.5) compared to HC (mean = 26.2), this difference was not statistically significant in our sample (*Z*(16) = −1.21, *p* = .23).

**TABLE 1:** Group‐wise comparisons of clinical pain metrics between healthy pain‐free controls and subjects with temporomandibular disorders (TMD)

Parameter^a^	Healthy controls (*n* = 6)	TMD subjects (*n* = 10)	*U* ^b^	*p* ^c^
Current face pain	0.0 (0.0)^a^	2.0 (1.3)	6.0	.005
Symptom severity scale	1.6 (1.1)	5.2 (3.2)	6.5	.022
Widespread Pain Index (WPI)	0.5 (0.5)	5.1 (3.9)	1.5	.002
Fibromyalgianess	2.2 (1.5)	10.3 (6.9)	2.0	.005
Pennebaker Inventory of Limbic Languidness (PILL)	8.0 (5.2)	16.1 (7.6)	10.5	.033
Brief Pain Inventory (BPI)	0.5 (1.2)	4.8 (2.1)	2.0	.004

^a^ Values are reported as the mean (standard deviation).

^b^ Nonparametric Mann–Whitney *U* test statistic.

^c^ *p*‐values correspond to group‐wise comparisons.

**FIGURE 2:**
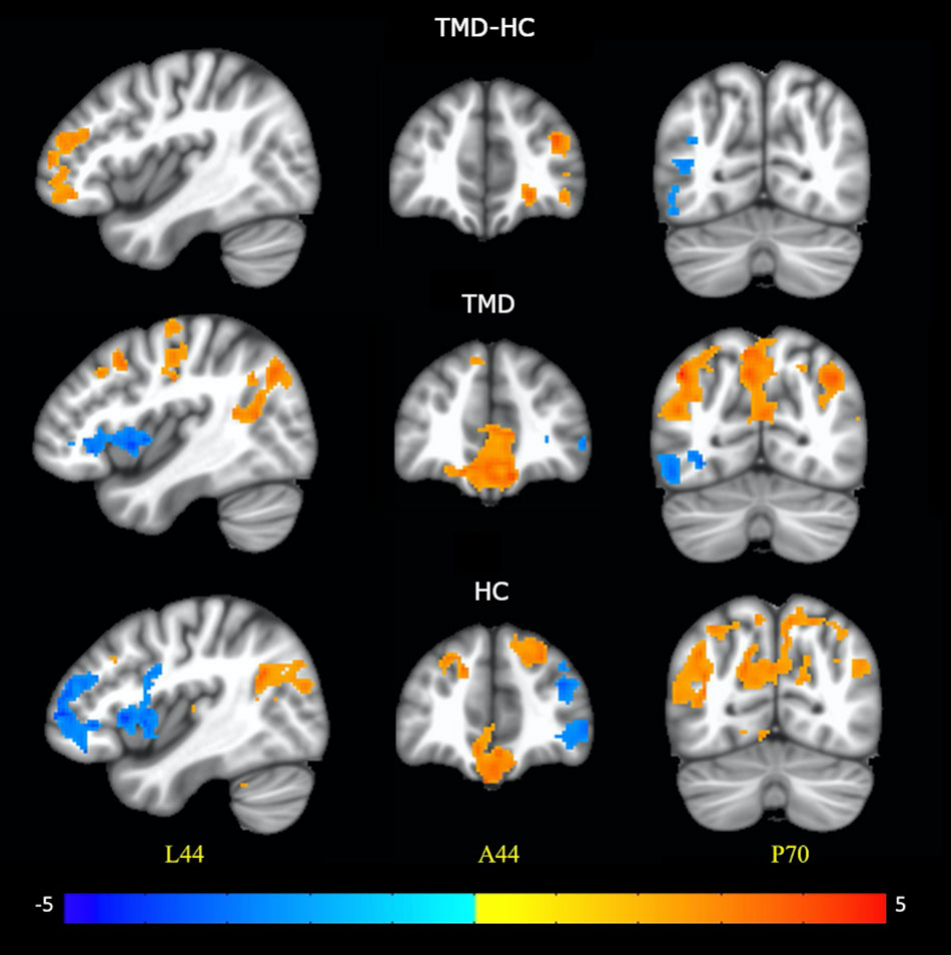
(Top) Significant (cluster‐level family‐wise error rate *α* < .05) differences in functional connectivity to the default mode network during the entire visual fMRI paradigm between subjects with temporomandibular disorders (TMD) and healthy control (HC) groups. One‐sample *t*‐test results for (middle) TMD and (bottom) HC groups. The color bar represents *t*‐test *z*‐scores. Slice locations are in MNI coordinates (L = left; A = anterior; P = posterior).

Group ICA yielded 11 artifact‐free ICs including those belonging to the default mode network (DMN), left and right frontoparietal networks, frontostriatal network, and social cognition network (Table 2). The DMN IC exhibited significant (*α* < .05) abnormally increased FC to right ventrolateral and dorsolateral prefrontal cortices in subjects with TMD compared to HC (Figure 2; Table 3). The TMD cohort exhibited reduced DMN FC with left occipitotemporal/inferior temporal cortex (Table 3). Subjects with TMD showed reduced FC between left frontoparietal network (attention and executive function) and higher order visual processing regions, including the extrastriate body area and lateral occipital complex in the left hemisphere. Subjects with TMD also exhibited abnormally increased FC between the frontostriatal network and cerebellum, and decreased FC between frontostriatal network and supplementary motor area compared to HC. Additionally, the social cognition network exhibited abnormally increased FC to inferior frontal and middle frontal cortices in subjects with TMD compared to HC.

**TABLE 2:** List of brain functional networks activated

Default mode
Higher order visual processing
Right frontoparietal
Left frontoparietal
Somatosensory and pain processing
Sensorimotor
Frontostriatal
Visuospatial attention
Anterior visual
Posterior visual
Social cognition

**TABLE 3:** Areas exhibiting significant differences in functional connectivity (family‐wise error rate [FWER]‐corrected *α* < .05) to specific resting state networks (group independent components) during the visual functional magnetic resonance imaging paradigm between subjects with temporomandibular disorders (TMD) and healthy controls (HC)

TMD vs. HC	Regions	Cluster peak z‐statistic	Cluster size (2 × 2 × 2 mm^3^ voxels)	Cluster detection threshold p‐values	Cluster‐level FWE rate α
Default mode network					
TMD > HC	Right hemisphere: dorsolateral and ventrolateral prefrontal cortex	3.86	4688	<.05	<.05
TMD < HC	Left occipitotemporal cortex	–3.26	4296	<.05	<.05
Left frontoparietal network					
TMD > HC	Right posterior parietal cortex	4.21	6532	<.05	<.05
TMD < HC	Left extrastriate body area and lateral occipital complex	–4.22	5048	<.05	<.05
Cognitive control network					
TMD > HC	Anterior cerebellar vermis	3.83	3776	<.05	<.05
TMD < HC	Supplementary motor area	–4.18	3928	<.05	<.05
Social cognition					
TMD > HC	Right inferior frontal and middle frontal gyrus	4.458	7632	<.05	<.05

*Note*: Regions were identified using brain atlases in AFNI. Cluster‐level *α* are obtained with the assumption the spatial autocorrelation function is Gaussian (see Section 2).

## 4 DISCUSSION

Sensory hypersensitivity is emerging as a key feature of nociplastic pain, which may drive clinical pain in many chronic overlapping pain conditions (Fitzcharles et al., 2021; Maixner et al., 2016). In this study, group ICA analysis of an unpleasant visual stimulus task during an fMRI paradigm revealed increased DMN FC to areas involved in attention, including dorsolateral and ventrolateral prefrontal cortices in TMD patients, possibly indicating increased attention to stimuli in TMD, consistent with prior work in chronic pain (Tracey & Bushnell, 2009; Villemure & Bushnell, 2009). Stronger activation of DMN regions associated with attention and salience was previously observed during a Stroop task in TMD compared to controls (Weissman‐Fogel et al., 2011). The DMN is typically suppressed during attention and executive functions, supporting the hypothesis that DMN‐associated dysfunction may relate to pain modulation. Indeed, DMN alterations have been associated with both chronic pain and pain‐evoked activity in healthy individuals (Alshelh et al., 2018). Resting‐state DMN alterations have been reported following gnathological treatment in TMD, suggesting that DMN‐associated changes may span rest, task‐evoked activation, and treatment response (Festa et al., 2021). The prefrontal cortex (PFC), in particular, is involved in executive function. Changes in grey matter, brain metabolites, and FC in the PFC have been reported in subjects with TMD as well as chronic back pain, myofascial pain, and fibromyalgia (Domin et al., 2021; Kucyi et al., 2014; Ong et al., 2019; Yin et al., 2020). Furthermore, transcranial direct current stimulation and repetitive transcranial magnetic stimulation targeting the dorsolateral PFC have shown some promise (Ong et al., 2019).

We also observed reduced FC in TMD compared to HC between the frontoparietal attention/executive function network and higher order visual processing areas, and between the cognitive control network and supplementary motor area. Motor dysfunction has been observed in TMD, including increased motor cortex activation (Weissman‐Fogel et al., 2011). Alterations in executive function and attention may be the result of pain hypervigilance or rumination (Broadbent et al., 2021; Michael & Burns, 2004). A recent review presents evidence for attentional bias to somatosensory stimuli in individuals with chronic pain (Broadbent et al., 2021), and pain rumination has been positively associated with FC in the medial PFC and other DMN regions in TMD (Kucyi et al., 2014). While more work is warranted, a growing body of literature suggests chronic pain is associated with widespread brain changes that may affect multisensory processing, attention, and executive function.

As a pilot study, we acknowledge limitations of sample size and primarily female participants. Future studies will expand these initial results to larger cohorts, additional sensory stimuli, and structural and metabolic changes to evaluate the central mechanisms associated with multisensory hypersensitivity in TMD. Larger studies will also enable the use of additional correlations with factors that could affect FC including clinical pain intensity and duration of TMD. We expect these and similar results from brain neuroimaging studies may be used to help differentiate pain mechanisms in TMD toward more personalized and tailored treatment.

## CONFLICT OF INTEREST STATEMENT

Dr. Harte has received research support from Aptinyx and Arbor Medical Innovations, and consultation fees from Aptinyx, Memorial Slone Kettering Cancer Center, Indiana University, and University of North Carolina—Chapel Hill, not related to the present work.

### PEER REVIEW

The peer review history for this article is available at https://publons.com/publon/10.1002/brb3.2916.

## Data Availability

Data will be made available upon request after a data sharing agreement is executed.
